# Folic acid, either solely or combined with L‐citrulline, improves NO signaling and ameliorates chronic hypoxia‐induced pulmonary hypertension in newborn pigs

**DOI:** 10.14814/phy2.15096

**Published:** 2021-11-11

**Authors:** Matthew Douglass, Anna Dikalova, Mark R. Kaplowitz, Yongmei Zhang, Gary Cunningham, Marshall Summar, Candice D. Fike

**Affiliations:** ^1^ Department of Pediatrics University of Utah Health Salt Lake City Utah USA; ^2^ Department of Medicine Vanderbilt University Medical Center Nashville Tennessee USA; ^3^ Division of Genetics and Metabolism Children’s National Medical Center Washington District of Columbia USA

**Keywords:** pulmonary resistance arteries, superoxide, uncoupled eNOS

## Abstract

Concomitant with developing pulmonary hypertension (PH), newborn piglets exposed to chronic hypoxia develop pulmonary vascular NO signaling impairments. PH is reduced and NO signaling is improved in chronically hypoxic piglets treated with the NO‐arginine precursor, L‐citrulline. Folic acid positively impacts NO signaling. We evaluated whether the effect on NO signaling and PH is greater using co‐treatment with folic acid and L‐citrulline than either alone. From day 3 to day 10 of hypoxia, piglets were treated solely with folic acid, solely with L‐citrulline, or co‐treated with both. Catheters were placed to measure in vivo hemodynamics. NO production was measured in vitro in dissected pulmonary arteries. Compared to normoxic piglets, pulmonary vascular resistance (PVR) was elevated and NO production was reduced in untreated hypoxic piglets. Regardless of treatment strategy, PVR was less in all three treated groups of hypoxic piglets when compared to the untreated hypoxic group. In addition, for all three groups of treated hypoxic piglets, NO production was higher than the untreated group. Improvements in PVR and NO production did not differ between piglets co‐treated with folic acid and L‐citrulline and those treated solely with either. Thus, the impact on NO production and PVR was not augmented by combining folic acid and L‐citrulline treatments. Nonetheless, treatment with folic acid, either singly or when combined with L‐citrulline, increases NO production and inhibits PH in chronically hypoxic newborn piglets. Folic acid merits consideration as a therapy for PH in human infants with chronic heart and lung conditions that are associated with chronic hypoxia.

## Graphical abstract

We report for the first time that oral administration of the B vitamin, folic acid, either solely, or when combined with the amino acid, L‐citrulline, ameliorated the development of pulmonary hypertension (PH) in a newborn piglet animal model. We also provide data showing that the mechanistic impact of folic acid is due to improving pulmonary vascular NO signaling. Thus, folic acid merits further investigation as a potential treatment for human infants with PH due to cardiopulmonary conditions associated with chronic hypoxia.

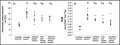

## 1 INTRODUCTION

Infants with chronic lung and heart disorders can develop pulmonary hypertension (PH), a devastating condition with few and often ineffective therapies (Abman et al., 2016). It is well accepted that hypoxia can contribute to the development of PH in these infants. Over the past few decades, a number of investigators have explored the possibility that dysregulation of the nitric oxide (NO) pathway contributes to many types of PH, including PH associated with hypoxia (Fike et al., 2004; Klinger et al., 2013; Tabima et al., 2012). Moreover, studies performed in animal models have provided evidence that targeting the NO pathway can be an effective way to treat PH (Ahmed et al., 2014; Chalupsky et al., 2015; Mitani et al., 1997; Sasaki et al., 2004; Schreiber et al., 2017).

For example, we previously found that concomitant with increases in NO production, chronic hypoxia‐induced PH was ameliorated in newborn piglets receiving oral treatment with the L‐arginine–NO precursor, L‐citrulline (Ananthakrishnan et al., 2009; Fike et al., 2015). However, it is notable that NO produced by the hypoxic animals treated with L‐citrulline remained less than levels measured in normoxic control animals (Ananthakrishnan et al., 2009; Fike et al., 2015). Moreover, the pulmonary vascular resistance (PVR) measured in the L‐citrulline‐treated hypoxic animals remained elevated above levels of PVR measured in normoxic control animals (Ananthakrishnan et al., 2009; Fike et al., 2015).

It is possible that higher doses of L‐citrulline might further reduce PVR and enhance NO production in vivo in hypoxic piglets. However, we recently performed in vitro studies and found that L‐citrulline dose‐dependently increases arginase activity in hypoxic piglet pulmonary artery endothelial cells (Douglass et al., 2021). This latter finding is concerning because arginase can decrease NO production by competing with eNOS for the substrate, L‐arginine (Akashi et al., 2001; Morris, 2009). Indeed, in part because of its negative impact on NO production, it has been suggested that arginase contributes to a number of different cardiovascular disorders, including PH (Kao et al., 2015; Nara et al., 2015; Xu et al., 2004). Thus, we were concerned that progressively increasing L‐citrulline would elevate arginase activity to levels adversely impacting NO signaling. Another issue is that the limited solubility of L‐citrulline means that in order to give a higher dose of L‐citrulline as an oral liquid therapy, a larger fluid volume must be given. Newborns with heart and lung diseases sometimes develop pulmonary edema when given high fluid volumes. To avoid potential adverse consequences from using progressively higher volumes and doses of L‐citrulline, we reasoned that alternate treatments that improve NO signaling should be pursued.

Folic acid is a water‐soluble B vitamin that has been shown to positively impact a number of aspects of NO signaling (Stanhewicz & Kenney, 2017). The high solubility of folic acid means that minimal fluid volumes are needed to administer even large doses as an oral liquid therapy. In this study, we wanted to determine if oral treatment with folic acid, given solely or when combined with L‐citrulline, would improve NO signaling and ameliorate PH in newborn piglets exposed to chronic hypoxia. We also wanted to determine whether combining oral treatments of L‐citrulline and folic acid would provide a more efficacious treatment than giving either therapy alone.

## 2 METHODS

### 2.1 Animal care and in vivo hypoxia model

All animal protocols were approved by the Institutional Animal Care and Use Committee of the University of Utah Health. This animal facility is fully accredited by the Association for Assessment and Accreditation of Laboratory Animal Use. In addition, we adhered to National Institutes of Health guidelines for the use of experimental animals. In previous studies, we have shown that PVR does not differ between piglets obtained from a vendor and those raised by us in a room air environment (Fike & Kaplowitz, 1994, 1996). Hence, the control animals used in this study were piglets raised by the vendor and studied at day of life 12 (*n* = 8; *n* = 4 females, *n* = 4 males). All piglets used as hypoxic animals were raised by us in a normobaric hypoxic chamber for 9–10 days, from day of life two until day of life 11–12. Thus, the hypoxic animals were studied at a comparable postnatal age as the normoxic control animals. The oxygen content in the hypoxic chamber was 10%–12% O_2_; and the CO_2_ was kept at 3–6 Torr by absorption with soda lime.

### 2.2 Animals: chronic folic acid and/or L‐citrulline supplementation

We studied four groups of hypoxic animals: an untreated group of hypoxic piglets and three treated groups of hypoxic piglets. The untreated group received no treatment at all throughout the exposure to hypoxia (*n* = 11; *n* = 8 females, *n* = 3 males). Treated hypoxic piglets were given either sole therapy with oral folic acid (*n* = 7; *n* = 3 females, *n* = 4 males), sole therapy with oral L‐citrulline (*n* = 8; *n* = 4 females, *n* = 4 males), or were co‐treated with folic acid and L‐citrulline (*n* = 11; *n* = 6 females, *n* = 5 males). All treatments were started on the 3rd day of hypoxic exposure and continued for an additional 6–7 days of hypoxia. This reflects a rescue treatment, since PH develops when piglets are exposed to 3 days of hypoxia and worsens when hypoxic exposure is extended to 10 days (Fike & Kaplowitz, 1994, 1996). We chose to study a rescue treatment to approximate current clinical treatments, which are started after PH is diagnosed, rather than prophylactically. All piglets treated with folic acid, either as sole or co‐therapy, were given 5 mg/kg orally by syringe of a liquid preparation of folic acid in the morning of each day of treatment. All piglets treated with L‐citrulline, either as sole or co‐therapy, were given a total daily dose of 1.5 g/kg/d. The L‐citrulline was solubilized in sterile water and some of it (0.26 g/kg) was given orally twice a day with a syringe. An additional 1.0 g/kg was mixed in milk, which was consumed throughout the day.

### 2.3 Animals: in vivo hemodynamics

To preanesthetize the piglets, ketamine (15 mg/kg im) and acepromazine (2 mg/kg im) were given. The piglets were then sedated with intravenous pentobarbital. A tracheostomy was placed followed by insertion of a femoral arterial catheter. Samples of blood were obtained and the plasma was collected and frozen at −80°C for later assessment of folic acid or L‐citrulline and L‐arginine levels. Next, catheters were placed in order to measure cardiac output by the thermodilution technique. To do this, a thermistor was placed in the aortic arch via a left femoral arterial catheter and a catheter to use as an injection port was placed in the left ventricle via a left carotid artery. Systemic arterial pressure and left ventricular diastolic pressure were measured with the arterial catheters. Pulmonary arterial pressure was measured with a venous catheter that was placed via the jugular vein. Throughout all surgical procedures and hemodynamic measurements piglets were breathing room air. If the animal became apneic, which sometimes occurred when the piglet was given sedation, a piston‐type ventilator was used to ventilate the animal with room air at a tidal volume of 10–15 ml/kg, end‐expiratory pressure of 2–3 Torr, and a respiratory rate of 20–25 breaths/min. When the hemodynamic measurements were completed, additional anesthesia was given, heparin (1000 IU/kg iv) was administered, and the piglet was exsanguinated.

### 2.4 Right ventricular mass assessment

Fulton's index, the ratio of the weight of the right ventricular free wall (RV) to the weight of the left ventricular wall and septum (LV+S) was calculated to assess right ventricular mass. To do this, the heart was removed and the RV and LV+S were separated and weighed.

### 2.5 Pulmonary artery isolation

Small pulmonary arteries (≤300 µm) were dissected and used for cannulated artery studies, measuring levels of NO production or superoxide generation, or were immediately frozen in liquid nitrogen and stored at −80°C and later used for protein analysis by western blot technique.

### 2.6 Cannulated artery studies

Using our previously described technique, basal tone was established in cannulated and pressurized small pulmonary arteries by equilibration for 30–60 min at a transmural pressure that reflects in vivo pressure (Fike et al., 2011; Fike & Kaplowitz, 1994, 1996). Contraction to the thromboxane mimetic, U46619, was assessed to check for viability. A functional endothelium was assessed in arteries from normoxic control piglets, by testing responses to acetylcholine, ACh (10^−6^ M). Because hypoxic arteries constrict to ACh, responses to the calcium ionophore A23187 were used to test for a functional endothelium in hypoxic arteries (Fike et al., 2002).

Studies to evaluate responses to the NO donor, S‐nitroso‐N‐acetyl‐penicillamine (SNAP) were then performed. For these studies, endothelin (10^−10^ to 10^−9^ M) or U46619 (10^−9^ to 10^−8^ M) was added to the reservoir to elevate tone by 40%–50%. Next, cumulative doses of SNAP, 10^−9^ to 10^−5^ M, were added to the reservoir.

### 2.7 NO measurements by electron spin resonance (ESR)

Small pulmonary arteries (≤300 μm diameter) were incubated for 60 min at 37°C in 1.5 ml of Krebs/HEPES buffer containing 200 μmol/L iron diethyldithiocarbamate (Fe[DETC]_2_) and 10 μmol/L A‐23187. Previously described ESR methods were then used to detect the NO‐Fe[DETC]_2_ complex for the incubated arteries (Dikalova et al., 2010). Vessel protein content was determined by the Bradford assay and used to normalize the ESR values.

### 2.8 Superoxide measurements by lucigenin (N,N’‐Dimethyl‐9.9’‐biacridinium dinitrate) enhanced chemiluminescence

Using a modification of previously described methods (Fike et al., 2008), small pulmonary arteries (≤300 µM diameter) were minced and then incubated in Krebs Hepes buffer for 20–30 min at 37°C. After spinning in a centrifuge and discarding the supernatant, a pellet of the minced vessels was collected. To measure background relative light units (RLUs), a vial containing 2 ml of Krebs Hepes plus 5 µM lucigenin was placed in a luminometer (Titertek Instruments,). Next, the vessel pellet was added to the vial and RLUs readings were repeated. The pellet was collected and dried. The background RLUs were subtracted from the RLUs measured in the presence of the vessel pellet and normalized to the pellet dry weight.

### 2.9 Immunoblot analysis of total eNOS and eNOS dimers and monomers

We applied published methods (Fike et al., 2004) to frozen small pulmonary artery (≤300 μm diameter) samples to assess eNOS and eNOS dimers/monomers (eNOS antibody was from BD‐Transduction Laboratory) by immunoblot technique. Membranes were developed using enhanced chemiluminescence reagents (PerkinElmer). An iBright FL1500 imaging system (Thermo Fisher) was used to capture the chemiluminescent signal. The iBright Analysis Software was used to quantify the bands for each protein. The membranes were washed and stripped, after which a similar procedure was followed to reprobe the membranes for β‐actin (1:100,000, Sigma‐Aldrich).

### 2.10 Plasma amino acid analysis

To measure plasma concentrations of citrulline and arginine, we used previously described methods and performed amino acid analysis on protein‐free extracts (Ananthakrishnan et al., 2009). A Hitachi L8800 amino acid analyzer was used to separate the amino acids by cation exchange chromatography.

### 2.11 Plasma folic acid analysis

Plasma folic acid concentrations were measured by ARUP Laboratories, Salt Lake City, UT.

### 2.12 Statistical analysis

Data are presented as mean ± SD. Data were compared between groups by one‐way ANOVA with Fisher's protected least significant differences post hoc comparison test (Meier, 2006). A *p* value <0.05 was considered significant.

## 3 RESULTS

**FIGURE 1:**
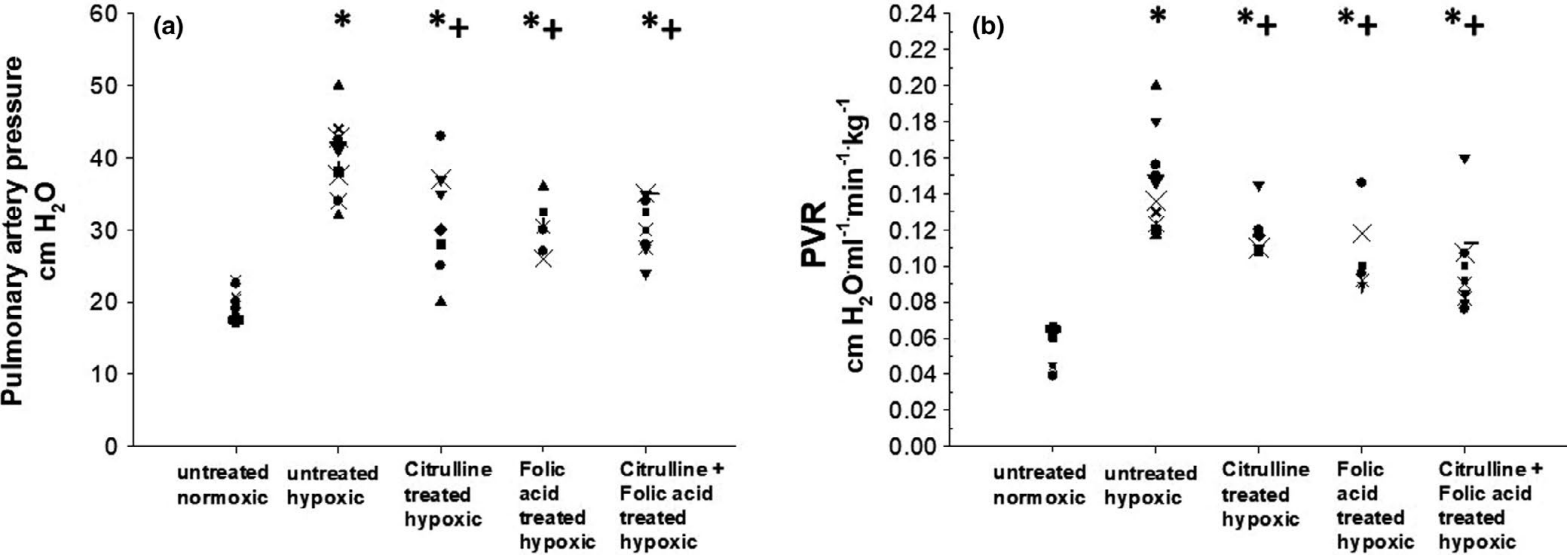
(a) Pulmonary artery pressure in control (normoxic) piglets (*n* = 8), untreated chronically hypoxic piglets (*n* = 11), and chronically hypoxic piglets receiving sole treatment with oral L‐citrulline (*n* = 8), sole treatment with oral folic acid (*n* = 7), or co‐treatment with L‐citrulline plus oral folic acid (*n* = 11). *Different from control (normoxic); ^+^different from untreated chronic hypoxic; *p* < 0.05; ANOVA with post hoc comparison test. (b) Pulmonary vascular resistance (PVR) in control (normoxic) piglets (*n* = 8), untreated chronically hypoxic piglets (*n* = 11), and chronically hypoxic piglets receiving sole treatment with oral L‐citrulline (*n* = 8), sole treatment with oral folic acid (*n* = 7), or co‐treatment with L‐citrulline plus oral folic acid (*n* = 11). *Different from control (normoxic); ^+^different from untreated chronic hypoxic; *p* < 0.05; ANOVA with post hoc comparison test

The untreated group of chronically hypoxic piglets had higher measurements of pulmonary arterial pressures when compared to measurements of pulmonary arterial pressure obtained in the normoxic control animals (Figure 1a). In addition, values of pulmonary arterial pressure were higher in all three treatment groups of hypoxic piglets, that is, hypoxic piglets treated with folic acid or L‐citrulline either as sole or combined therapy, when compared to values of pulmonary arterial pressure measured in the normoxic piglets (Figure 1a). There was no difference between pulmonary arterial pressures measured in the three treatment groups of hypoxic animals (Figure 1a). Notably, regardless of treatment strategy, all three treated groups of hypoxic piglets had values of pulmonary arterial pressure that were less than those measured in the untreated group of hypoxic piglets (Figure 1a).

Findings for PVR (Figure 1b) paralleled those for pulmonary arterial pressures (Figure 1a). That is, all four groups of hypoxic piglets had greater values of PVR than were measured in the group of the normoxic control piglets (Figure 1b). Measurements of PVR were similar for the three treated groups of hypoxic animals, regardless of treatment strategy (Figure 1b). Of note, all three treated groups of hypoxic piglets had values of PVR that were less than values of PVR measured in the untreated group of hypoxic animals (Figure 1b).

As summarized in the Table 1, aortic pressure measurements and measurements of arterial pH, arterial PO_2_, and arterial pco_2_ did not differ between any of the five groups of piglets. All four groups of hypoxic piglets had lower values of cardiac output, higher measurements of LVEDP, and greater values for right ventricular mass than those measured in the normoxic control piglets (Table 1). Measurements of cardiac output, LVEDP, and right ventricular mass were similar for all four hypoxic groups (Table 1).

**TABLE 1:** Data for normoxic (control), chronic hypoxic, and orally treated (L‐citrulline, folic acid, or L‐citrulline plus folic acid) chronic hypoxic piglets

Treatment group	LVEDP Cm H_2_O	Cardiac output ml.min^−1^.kg^−1^	Aortic pressure Cm H_2_O	RV/LV + S	pH	PaO_2_ Torr	PaCO_2_ Torr
Controls (normoxic) *N* = 8	5.4 ± 1.1	298 ± 65	73 ± 6	0.36 ± 0.05	7.40 ± 0.04	72 ± 7	32 ± 4
Chronic hypoxic *N* = 11	7.6 ± 0.7*	227 ± 37*	80 ± 7	0.8 ± 0.12*	7.44 ± 0.05	77 ± 6	30 ± 5
L‐citrulline chronic hypoxic *N* = 8	7.3 ± 0.5*	197 ± 62*	81 ± 10	0.82 ± 0.08*	7.40 ± 0.07*	75 ± 7	33 ± 5
Folic acid chronic hypoxic *N* = 7	7.3 ± 1.0*	225 ± 75*	79 ± 9	0.83 ± 0.09*	7.40 ± 0.07*	73 ± 16	34 ± 5
L‐citrulline + folic acid chronic hypoxic *N* = 11	7.1 ± 0.5*	242 ± 44*	80 ± 9	0.78 ± 0.13*	7.41 ± 0.06	72 ± 10	34 ± 10

Note

Values are means ± SD.

Abbreviations: LVEDP, left ventricular end‐diastolic pressure. RV/LV+S, right ventricle weight/left ventricle plus septum weight.

* Different from normoxic controls; *p* < 0.05; ANOVA with post hoc comparison test.

**FIGURE 2:**
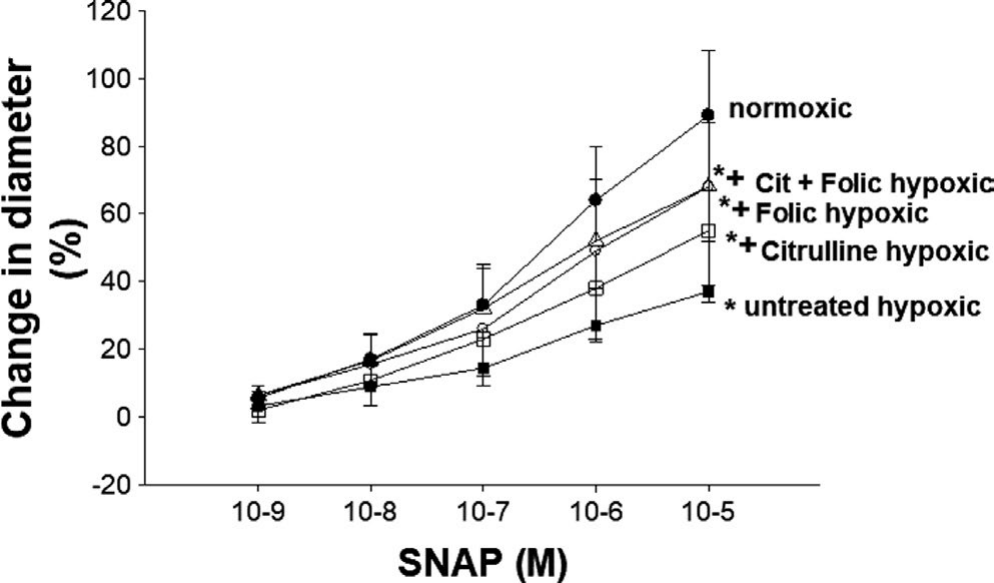
Responses to the NO donor, SNAP, in small pulmonary arteries from control (piglets [*n* = 8], untreated chronically hypoxic piglets [*n* = 11], and chronically hypoxic piglets receiving sole treatment with oral L‐citrulline [*n* = 8], sole treatment with oral folic acid [*n* = 7], or co‐treatment with L‐citrulline plus oral folic acid [*n* = 11]). *Different from control (normoxic); ^+^different from untreated chronic hypoxic; *p* < 0.05; ANOVA with post hoc comparison test

Dilation in response to the NO donor, SNAP, was less in pulmonary arteries from all four groups of hypoxic piglets than the degree of dilation to SNAP measured in small pulmonary arteries from the normoxic animals (Figure 2). However, the dilator response to SNAP was greater in pulmonary arteries from all three treated groups of hypoxic piglets when compared to the degree of dilation measured in pulmonary arteries from the untreated group of hypoxic piglets (Figure 2). The pulmonary artery dilator responses to SNAP did not differ between the three treated groups of hypoxic piglets (Figure 2).

**FIGURE 3:**
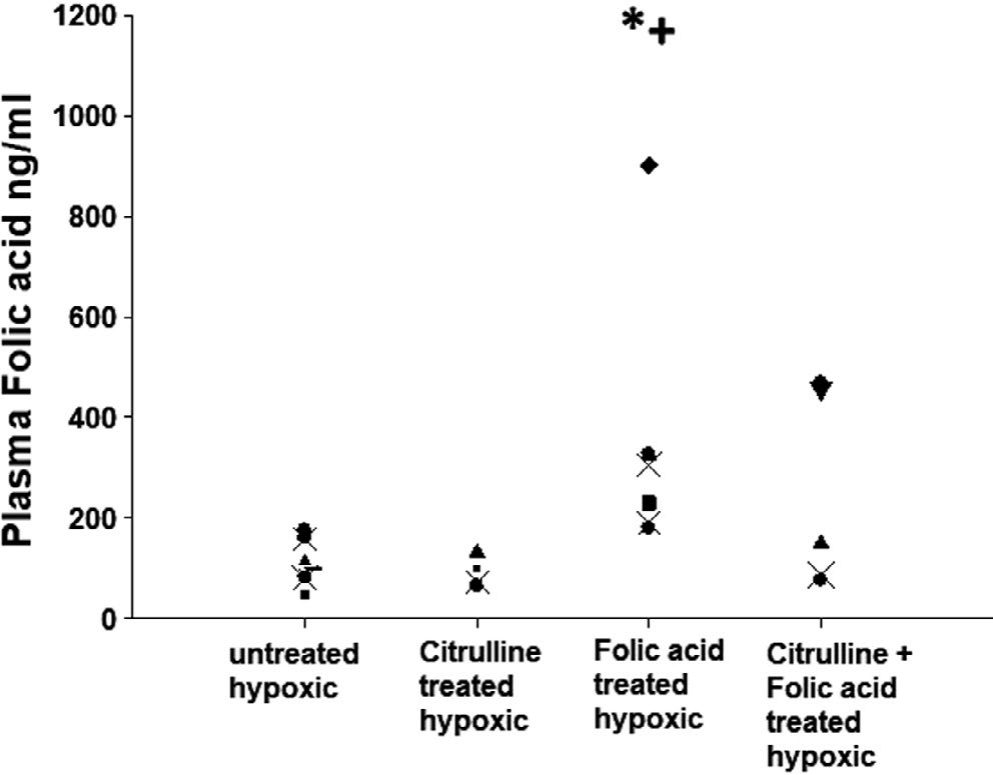
Folic acid plasma levels in untreated chronically hypoxic piglets (*n* = 11), and chronically hypoxic piglets receiving sole treatment with oral L‐citrulline (*n* = 4), sole treatment with oral folic acid (*n* = 7), or co‐treatment with L‐citrulline plus oral folic acid (*n* = 5). *Different from control (normoxic); ^+^different from untreated chronic hypoxic; *p* < 0.05; ANOVA with post hoc comparison test

Plasma folic acid levels were undetectable in normoxic control piglets, reflecting the fact that maternal sow milk does not contain folic acid. All groups of hypoxic animals had measurable levels of plasma folic acid since they were fed sow milk replacer to which folic acid has been added by the manufacturer (Figure 3). Of note, plasma folic acid levels did not differ between hypoxic piglets treated solely with folic acid and those co‐treated with folic acid and L‐citrulline (Figure 3). Nor did plasma folic acid levels differ between the untreated group of hypoxic piglets, hypoxic piglets treated solely with L‐citrulline, and those co‐treated with folic acid and L‐citrulline (Figure 3). Hypoxic piglets treated solely with folic acid had greater plasma folic acid levels than levels measured in either untreated hypoxic piglets or those treated solely with L‐citrulline (Figure 3).

**FIGURE 4:**
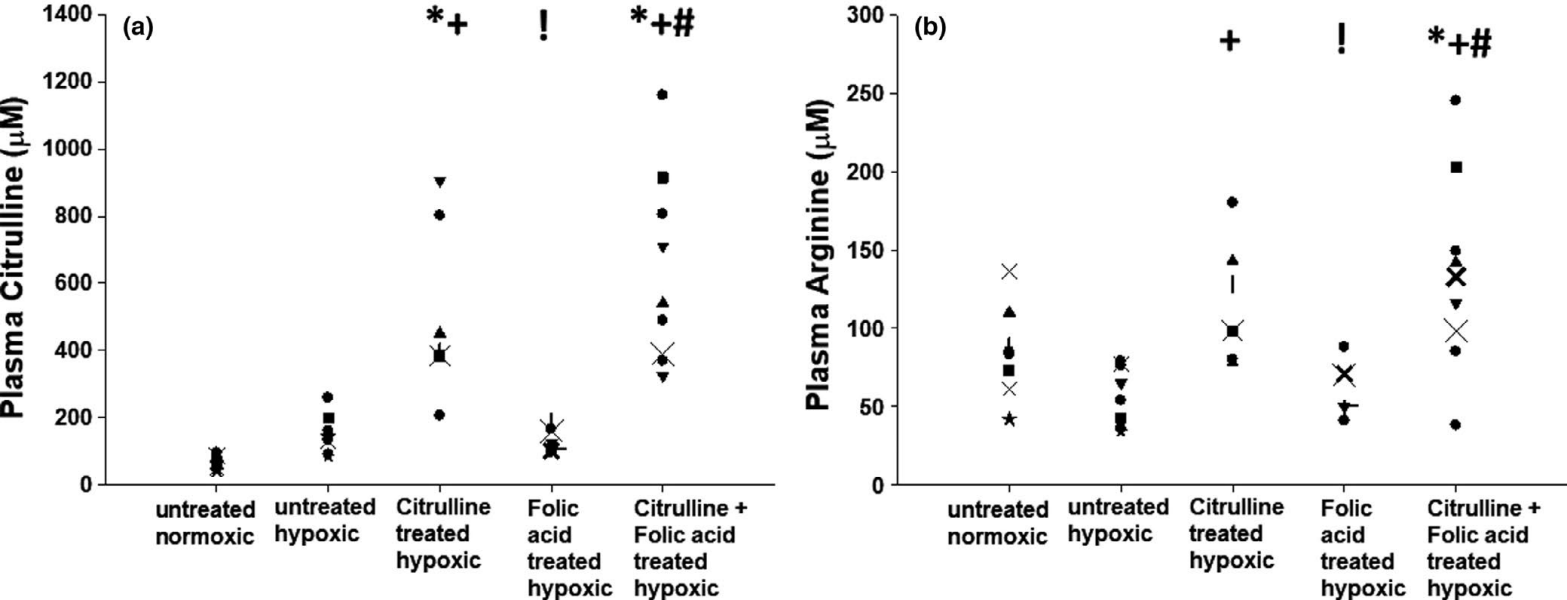
(a) Plasma citrulline levels in control (normoxic) piglets (*n* = 8), untreated chronically hypoxic piglets (*n* = 8), and chronically hypoxic piglets receiving sole treatment with oral L‐citrulline (*n* = 7), sole treatment with oral folic acid (*n* = 7), or co‐treatment with L‐citrulline plus oral folic acid (*n* = 9). *Different from control (normoxic); ^+^different from untreated chronic hypoxic; ^!^different from sole treatment with L‐citrulline; ^#^different from sole treatment with folic acid; *p* < 0.05; ANOVA with post hoc comparison test. (b) Plasma arginine levels in control (normoxic) piglets (*n* = 8), untreated chronically hypoxic piglets (*n* = 8), and chronically hypoxic piglets receiving sole treatment with oral L‐citrulline (*n* = 7), sole treatment with oral folic acid (*n* = 7), or co‐treatment with L‐citrulline plus oral folic acid (*n* = 9). *Different from control (normoxic); ^+^different from untreated chronic hypoxic; ^!^different from sole treatment with L‐citrulline; ^#^different from sole treatment with folic acid; *p* < 0.05; ANOVA with post hoc comparison test

There was no difference in plasma levels of L‐citrulline between the groups of normoxic control piglets, untreated hypoxic piglets, and hypoxic piglets treated solely with folic acid (Figure 4a). Levels of plasma L‐citrulline were higher in the two groups of hypoxic piglets treated with L‐citrulline, either solely or in combination with folic acid, than levels measured in the other three groups of animals (Figure 4a). There was no difference in plasma arginine levels measured in the groups of normoxic control piglets, untreated hypoxic piglets, and hypoxic piglets treated solely with folic acid (Figure 4b). Arginine levels were similar for hypoxic piglets treated solely with L‐citrulline and those co‐treated with folic acid and L‐citrulline (Figure 4b).

**FIGURE 5:**
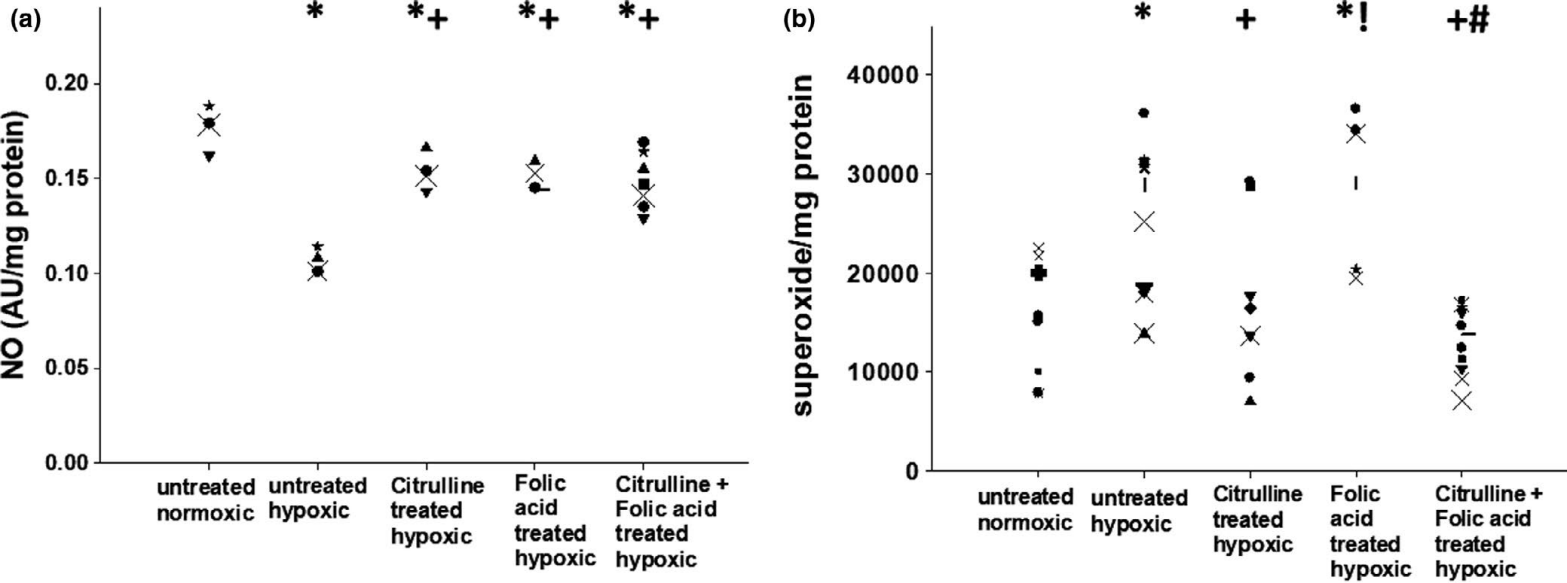
(a) Nitric oxide (NO) production by small pulmonary arteries from control (normoxic) piglets (*n* = 4), untreated chronically hypoxic piglets (*n* = 4), and chronically hypoxic piglets receiving sole treatment with oral L‐citrulline (*n* = 4), sole treatment with oral folic acid (*n* = 4), or co‐treatment with L‐citrulline plus oral folic acid (*n* = 7). *Different from control (normoxic); ^+^different from untreated chronic hypoxic; *p* < 0.05; ANOVA with post hoc comparison test. (b) Superoxide production by small pulmonary arteries from control piglets (*n* = 8), untreated chronically hypoxic piglets (*n* = 11), and chronically hypoxic piglets receiving sole treatment with oral L‐citrulline (*n* = 8), sole treatment with oral folic acid (*n* = 7), or co‐treatment with L‐citrulline plus oral folic acid (*n* = 11). *Different from control (normoxic); ^+^different from untreated chronic hypoxic; ^!^different from sole treatment with L‐citrulline; ^#^different from sole treatment with folic acid; *p* < 0.05; ANOVA with post hoc comparison test

Small pulmonary arteries from control piglets had higher levels of NO production when compared to levels measured in small pulmonary arteries from any of the four groups of hypoxic animals (Figure 5a). Small pulmonary arteries from all three treated groups of hypoxic piglets had higher levels of NO production than levels measured in small pulmonary arteries from the untreated group of hypoxic piglets (Figure 5a). Values for NO production were similar for all three treated groups of hypoxic animals (Figure 4a).

Values for superoxide generation were less (Figure 5b) in small pulmonary arteries from normoxic control piglets when compared to values measured in small pulmonary arteries from either the untreated group of hypoxic piglets or those treated solely with folic acid (Figure 5b). Superoxide generation did not differ between small pulmonary arteries from untreated hypoxic animals and those treated solely with folic acid (Figure 5b). The amount of superoxide generated by small pulmonary arteries from hypoxic animals treated solely with L‐citrulline and from hypoxic animals co‐treated with folic acid plus L‐citrulline were similar to levels from the normoxic control group and less than levels generated by small pulmonary arteries from the untreated hypoxic animals and the hypoxic animals treated solely with folic acid (Figure 5b).

**FIGURE 6:**
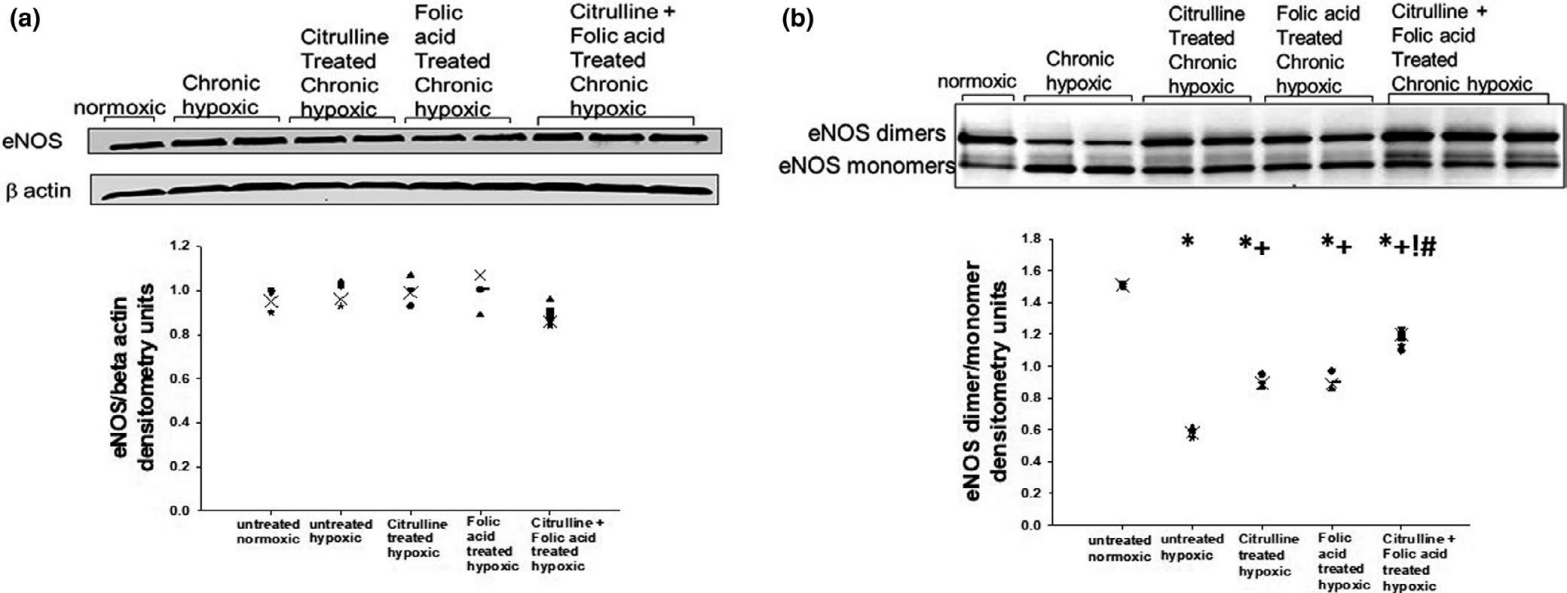
(a) A representative western blot for eNOS expression with corresponding densitometry shows that eNOS expression was similar in small pulmonary arteries from all groups of piglets (*n* = 4 control piglets, *n* = 4 chronically hypoxic piglets, *n* = 4 chronically hypoxic piglets receiving sole treatment with oral L‐citrulline, *n* = 4 chronically hypoxic piglets receiving sole treatment with oral folic acid, and *n* = 6 chronically hypoxic piglets receiving co‐treatment with L‐citrulline plus oral folic acid). (b) A representative western blot for eNOS dimer‐to‐monomer ratios with corresponding densitometry in small pulmonary arteries from all groups of piglets (*n* = 4 control piglets, *n* = 4 chronically hypoxic piglets, *n* = 4 chronically hypoxic piglets receiving sole treatment with oral L‐citrulline, *n* = 4 chronically hypoxic piglets receiving sole treatment with oral folic acid, and *n* = 6 chronically hypoxic piglets receiving co‐treatment with L‐citrulline plus oral folic acid). *Different from control (normoxic); ^+^different from untreated chronic hypoxic; ^!^different from sole treatment with L‐citrulline; ^#^different from sole treatment with folic acid; *p* < 0.05; ANOVA with post hoc comparison test

All five groups of piglets had similar total eNOS protein amounts (Figure 6a). The untreated group of hypoxic piglets had lower eNOS dimer‐to‐monomer ratios compared to normoxic control piglets (Figure 6b). eNOS dimer‐to‐monomer ratios were higher for all three treatment groups of hypoxic animals than for the untreated group of hypoxic piglets (Figure 6b). The group of hypoxic piglets co‐treated with folic acid plus L‐citrulline had higher eNOS dimer‐to‐monomer ratios than the hypoxic piglets treated with either folic acid or L‐citrulline alone (Figure 6b).

## 4 DISCUSSION

Findings in this study are consistent with those in our previous studies (Ananthakrishnan et al., 2009; Fike et al., 2015), and add to the body of evidence showing that impaired pulmonary vascular NO signaling contributes to the development of chronic hypoxia‐induced PH (Fike et al., 2004; Klinger et al., 2013; Tabima et al., 2012). Also consistent with findings in our previous studies (Ananthakrishnan et al., 2009; Fike et al., 2015), we show that treatment with oral L‐citrulline improved NO signaling and ameliorated PH in chronically hypoxic newborn piglets. An important new finding in this study is that when given solely or when combined with L‐citrulline, oral treatment with folic acid also improved NO signaling and ameliorated PH in chronically hypoxic newborn piglets.

In this study, we treated the hypoxic piglets with the highest dose of L‐citrulline that we have previously used to test for efficacy in inhibiting chronic hypoxia‐induced PH (Ananthakrishnan et al., 2009; Fike et al., 2015). We chose this dosing strategy because we previously found that the impact on PVR was greater in chronically hypoxic piglets treated with a higher (1.5 g/kg/d) than a lower (0.26–0.52 g/kg/d) L‐citrulline dosing strategy. Specifically, when compared to values in the untreated group of chronically hypoxic piglets, we previously found that the lower dose L‐citrulline strategy reduced PVR by approximately 17%; whereas the higher dose L‐citrulline strategy reduced PVR by approximately 33%. Moreover, in our previous study, we also found that piglets treated with the higher, but not the lower dose, L‐citrulline strategy had measurements of right ventricular hypertrophy (RVH) and LVEDP that were less than those measured in the untreated group of chronically hypoxic piglets. Despite the use of the higher dose L‐citrulline treatment strategy, in this study we found that neither RVH nor LVEDP was reduced below values measured in the untreated group of chronically hypoxic piglets. However, it should be noted that in this study we found that values of PVR measured in the L‐citrulline‐treated group of chronically hypoxic piglets were approximately 17.5% below values in the untreated group. It is not clear why the higher dose treatment strategy had less of an impact on PVR in chronically hypoxic piglets in this study than our previous study. One contributing factor could be that the previous study was performed at sea level while piglets in this study were raised at high altitude. Nonetheless, it seems likely that, similar to findings for the lower dose L‐citrulline‐treated group in our previous study, the reduction in PVR found in the L‐citrulline‐treated group in this study was not sufficient enough or sustained for a long enough period of time to reduce the strain on the right heart and thereby lead to reductions in RVH and LVEDP.

We considered the possibility that treatment with even higher doses of L‐citrulline might be even more efficacious. However, we were concerned with the possibility of uncovering adverse events from using higher L‐citrulline doses. One possible adverse consequence, raised by our recent in vitro findings (Douglass et al., 2021), is that progressively increasing the dose of L‐citrulline would elevate arginase activity to levels adversely impacting NO signaling. We were also mindful that newborns with chronic lung and heart diseases sometimes develop pulmonary edema when given high fluid volumes. The limited solubility of L‐citrulline means that in order to give a higher dose of L‐citrulline as an oral liquid therapy, a larger fluid volume must be given. Older patients can be given oral drugs in non‐liquid formulations, such as powders and pills. However, because newborns can only be given oral medications that are formulated as liquids, the volume of drug becomes an important consideration.

The above concerns led us to evaluate folic acid as an alternate oral therapy for use in ameliorating chronic hypoxia‐induced PH. Because of its high solubility, minimal fluid volumes are needed to administer even large doses of folic acid as an oral liquid therapy. Folic acid has been shown to improve NO signaling by a number of mechanisms, including increasing the effectiveness of the eNOS cofactor, BH_4_ (Stanhewicz & Kenney, 2017), positively impacting the phosphorylation of eNOS (Taylor et al., 2013), and by re‐coupling eNOS (Chalupsky et al., 2015). Our finding that sole treatment with folic acid increased NO production and eNOS dimer‐to‐monomer ratios suggests that at least one of the mechanisms by which folic acid improves NO production in small pulmonary arteries from hypoxic piglets is via improving eNOS coupling.

A finding that surprised us was that the amount of superoxide produced by small pulmonary arteries from the hypoxic piglets treated solely with folic acid remained similar to superoxide levels produced by pulmonary arteries from the untreated group of hypoxic piglets. Based on the evidence that folic acid can re‐couple eNOS, we would anticipate a reduction in superoxide generation to accompany the increase in NO production that we found. This is because, in the homodimer or coupled state, electrons are transferred from the eNOS reductase domain to the oxygenase domain and NO is produced; whereas when eNOS becomes uncoupled, electrons are diverted to molecular oxygen‐producing superoxide instead of NO (Stuehr et al., 2001; Tabima et al., 2012).

It is possible that an adverse consequence of folic acid therapy might be involved with our superoxide findings. It is known that excessive folic acid supplementation can cause unmetabolizable folic acid to accumulate in the blood (Koseki et al., 2020; Patanwala et al., 2014; Sweeney et al., 2009). If concentrations of unmetabolized folic acid reach high levels in cells, metabolic disorders of the folate and methionine metabolic cycles can be induced, leading to the intracellular formation of unmetabolizable homocysteine, which is a pro‐oxidant (Koseki et al., 2020). In particular, homocysteine has been shown to produce reactive oxygen species by undergoing self‐oxidation and by activating NADPH oxidase (Olszewski & McCully, 1993; Siow et al., 2006). Indeed, recent studies with Caenorhabditis elegans as an animal model showed that high dose folic acid supplementation increased intracellular levels of homocysteine, the reactive oxygen species, H_2_O_2_, and a lipid peroxidation marker, malondialdehyde, MDA (Koseki et al., 2020). Thus, it is possible that the amount of folic acid we used to treat the hypoxic piglets might have caused a degree of intracellular oxidative stress that was sufficient to counteract any potential suppression of superoxide generation from eNOS re‐coupling.

The dose of folic acid that we used was based on studies performed by others (Chalupsky et al., 2015). Specifically, using a prophylactic treatment strategy, that is, starting treatment on the first day of hypoxic exposure, another group of investigators found that a dose of 5 mg/kg/d of folic acid protected adult mice from developing PH when exposed to 2 weeks of hypoxia (10% O_2_) (Chalupsky et al., 2015). We add to the literature by showing the ability to inhibit PH using a folic acid treatment strategy that better approximates current clinical strategies, that is, initiating treatments after the onset of PH. Notably, both studies found that folic acid improved eNOS dimer‐to‐monomer ratios in pulmonary arteries of the hypoxic animals. However, unlike our findings in small pulmonary arteries from chronically hypoxic piglets, superoxide levels measured in pulmonary arteries from folic acid‐treated chronically hypoxic mice were reduced below levels measured in untreated chronically hypoxic mice (Chalupsky et al., 2015). We are uncertain as to the reason for the different impact of folic acid on pulmonary artery superoxide generation between studies. However, differences in species, postnatal ages, size of pulmonary artery used for evaluation, and dosing modality might have contributed. For example, adult mice received folic acid by dietary supplementation so that the amount of drug received would have been dependent on total daily dietary intake (Chalupsky et al., 2015); whereas piglets were administered a once daily dose of folic acid orally by syringe. Thus, the daily amounts of folic acid received and plasma levels of folic acid achieved in mice may have differed from those in newborn piglets.

In addition to use as a monotherapy that could serve as an effective alternate to L‐citrulline, we wanted to evaluate the effect of combining folic acid and L‐citrulline on chronic hypoxia‐induced PH. This approach seemed logical because these therapies have the potential to impact eNOS coupling by complementary mechanisms. L‐citrulline increases the bioavailability of L‐arginine, which is needed for optimal eNOS coupling (Gielis et al., 2011; Martasek et al., 1998). Folic acid improves the bio‐availability of BH_4_ (Chalupsky et al., 2015; Moens et al., 2008) which, in turn, promotes eNOS coupling by stabilizing eNOS in a dimeric configuration (Gielis et al., 2011; Xia et al., 1998). Indeed, in this study we found that when used as monotherapies, both folic acid and L‐citrulline increased the dimeric configuration of eNOS (Figure 6b). We also found that co‐treatment with folic acid and L‐citrulline had a greater impact on eNOS dimer‐to‐monomer ratios than did treatment with either alone (Figure 6b). Yet, small pulmonary artery NO production did not differ between piglets receiving co‐treatment with folic acid and L‐citrulline and those treated with either alone (Figure 5a). The reason for this finding remains unclear. However, since NO can be inactivated by interacting with reactive oxygen species (Gryglewski et al., 1986), it is possible that the amount of NO generated in small pulmonary arteries of piglets receiving folic acid, either as sole therapy or co‐treatment with L‐citrulline, was limited by interacting with superoxide. That is, our NO findings are likely to have been influenced by the failure of folic acid treatment to reduce superoxide to levels below those measured in the untreated group of hypoxic piglets. In addition, the relatively small number of animals studied and limitations with sensitivity of the assay could contribute to our inability to detect a difference in NO between the three treated groups of chronically hypoxic piglets.

Our finding that plasma folic acid levels did not differ between the untreated group of chronically hypoxic piglets, piglets treated solely with L‐citrulline, and piglets co‐treated with L‐citrulline and folic acid may have influenced some of our results and merits comment. That is, it is notable that groups of chronically hypoxic piglets who did not receive bolus doses of folic acid achieved plasma folic acid levels that were similar to those measured in chronically hypoxic piglets who received co‐treatment with L‐citrulline and bolus doses of folic acid. There are a couple of factors that likely contributed to the similarity of plasma folic acid levels in these groups. One factor is that sow milk replacer is supplemented with folic acid by the manufacturer so that, regardless of treatment strategy, all chronically hypoxic piglets received some folic acid. Another is that the total daily amount of milk consumed by the piglets was not regulated and could not be measured so that the total amount of folic acid received from their diet is not known and could vary considerably between animals. In addition, the enteral absorption of orally administered drugs, including folic acid, can be quite variable. Thus, it is possible that even though they did not receive bolus dose treatment with folic acid, piglets consumed enough sow milk to achieve folic acid levels similar to those measured in chronically hypoxic piglets who were administered bolus doses of folic acid. Moreover, it is possible that if the folic acid levels of the chronic hypoxia piglets co‐treated with L‐citrulline and folic acid had been greater than the untreated animals, that they would have demonstrated a greater reduction in their PVR and other parameters that are impacted by chronic hypoxia.

Other than the study with chronically hypoxic adult mice (Chalupsky et al., 2015), we are not aware of any other studies using animal models in which the impact on PH from the use of prolonged treatment with oral folic acid, either solely or combined with other oral therapies has been evaluated. Furthermore, although folic acid has been extensively evaluated as a therapy for a variety of cardiovascular disorders in adult humans (Moens et al., 2008; Wang et al., 2019), we do not know of any studies examining the efficacy of prolonged oral treatment with folic acid given as a sole or a co‐treatment for pulmonary vascular disease in humans of any age group. We are interested in oral therapies because PH in conditions associated with prolonged or intermittent hypoxia will require treatment for weeks, months, or possibly years. Long‐term intravenous therapies are associated with a number of adverse complications. Inhaled therapies can be poorly tolerated due to airway irritation and can be difficult to properly administer to newborns. Thus, effective, practical, and safe oral treatment strategies need to be developed for newborns.

Although folic acid has been considered to be safe, there has been a growing concern about appropriate dosing of folic acid. Mammals lack the necessary enzymes to synthesize folic acid de novo and therefore depend on dietary and supplemental folic acid to meet their biological needs (Lamers, 2011). Evidence strongly supports that adult women should take 400–800 micrograms of folic acid as a daily supplement during pregnancy to prevent fetal neural tube defects (Force et al., 2017). Higher doses of folic acid supplementation, up to 40–80 mg/day for 10 years, have been used as a therapy for a variety of cardiovascular disorders in adult humans without reports of adverse effects (Moens et al., 2008). Moreover, no adverse effects have been reported in adult mice treated with folic acid doses of 5–15 mg/kg/d for 2–4 weeks in studies evaluating the effectiveness of folic acid to improve a variety of cardiovascular diseases (Chalupsky et al., 2015; Gao et al., 2009; Siu et al., 2014).

There is much less experience using folic acid to treat cardiovascular disorders in children. Nonetheless, 8 weeks of treatment with 5 mg of oral folic acid reversed endothelial dysfunction without reports of adverse effects in children with type 1 diabetes (MacKenzie et al., 2006). As to newborns, although not a cardiovascular disorder, no adverse events were found when folic acid supplementation in the amounts of 100 micrograms/kg/d (Haiden et al., 2006) or 2 mg/d (Donato et al., 2000) were used to treat human infants with anemia of prematurity for time periods up to 6 weeks. Moreover, human infants receive folic acid from their diets. This is because human mothers receive folate in their diet such that human milk contains approximately 85–123 micrograms folate/liter and because folic acid, the synthetic form of folate, is added to formulas to simulate the concentration in human milk (Almeida et al., 2021; Hanson et al., 2012; Lamers, 2011). It has been estimated that human milk‐fed infants receive approximately 66 micrograms of folate per day (Lamers, 2011).

Notably, potential associations with negative health outcomes, such as augmenting cancer and influencing insulin resistance, have led to a growing controversy about the current use of folic acid as a food supplement (Choi et al., 2014; Lucock & Yates, 2009; Maruvada et al., 2020). In addition, the dosing needed and effectiveness of folic acid as a therapy for cardiovascular diseases have been inconsistent (Shirodaria et al., 2007; Zhou et al., 2018). Thus, there is growing awareness of the need for more research to assess the health effects of using folic acid in amounts exceeding current recommendations for upper limits (Field & Stover, 2018; Maruvada et al., 2020). It should be noted that the current upper limit of folic acid advised for use by the NIH Office of Dietary Supplements is 1000 micrograms per day for adults, 300–800 micrograms per day for children between 1 and 18 years of age, and is unknown for newborns and infants up to 12 months of age (Supplements, 2021). In this regard, it is important to acknowledge that the dose of folic acid used in this study was much higher than has been used as a dietary supplement or therapy in human infants. Moreover, it is important to realize that, due to the potential for adverse effects, the impact on development of chronic hypoxia‐induced PH might have been greater had we used lower doses of folic acid. Future studies evaluating the effect of different folic acid doses on the development of PH in newborn animal models would be of great interest.

Limitations of our study should be mentioned. One limitation is that we did not evaluate the effect of our treatment strategies on structural alterations in the lung parenchyma or the pulmonary circulation that is known to contribute to PH, such as pulmonary vascular remodeling. Nor did we perform studies to evaluate all the possible mechanisms by which the therapies might have impacted on NO signaling, such as alterations in phospho‐eNOS. Another limitation is that we used a newborn animal model of chronic hypoxia‐induced PH. The relevance of findings with this model to the human condition, and particularly to forms of neonatal PH that are not associated with exposure to hypoxia, is unknown. Moreover, although we performed studies in both female and male piglets, we did not perform enough studies to be able to evaluate the potential impact of sex on our findings. This is an important limitation worthy of future studies as there is an increasing amount of evidence supporting the influence of sex on the development of PH in adult humans.

In summary, findings in this study show that oral treatment with 5 mg/kg/d of folic acid, either solely or when combined with L‐citrulline, ameliorated the development of PH in a chronically hypoxic newborn piglet model. Of note, the 5 mg/kg/dose of folic acid improved NO signaling but did not have a beneficial impact on superoxide generation. It is possible, but remains to be studied, that lower doses of folic acid would have the ability to both inhibit PH and reduce superoxide generation in chronically hypoxic newborn piglets. Although use of folic acid as an oral therapy to treat human infants with PH in conditions associated with prolonged or intermittent hypoxia merits future consideration, more research is needed to guide the choice of dosing strategies that could be evaluated for safety and efficacy in clinical studies in human infants.

## CONFLICT OF INTEREST

C.D. Fike and M. Summar are listed on a patent application for the use of L‐citrulline as a therapeutic treatment for lung conditions.

## AUTHOR CONTRIBUTIONS

Matthew Douglass: Data acquisition, analysis, interpretation, and article writing; Anna Dikalova: Data acquisition, analysis, interpretation, and article writing; Mark R. Kaplowitz: Data acquisition and analysis; Yongmei Zhang: Data acquisition and analysis; Gary Cunningham: Data analysis; Marshall Summar: Hypothesis generation, data analysis, and interpretation; Candice D. Fike: Hypothesis generation, study design, data acquisition, analysis, interpretation, and article writing.
